# A benchmark for statistical microarray data analysis that preserves actual biological and technical variance

**DOI:** 10.1186/1471-2105-11-17

**Published:** 2010-01-11

**Authors:** Benoît De Hertogh, Bertrand De Meulder, Fabrice Berger, Michael Pierre, Eric Bareke, Anthoula Gaigneaux, Eric Depiereux

**Affiliations:** 1 Unité de Recherche en Biologie Moléculaire, Facultés Universitaires Notre-Dame de la Paix (F.U.N.D.P.), Rue de Bruxelles, 61, B-5000 Namur, Belgium

## Abstract

**Background:**

Recent reanalysis of spike-in datasets underscored the need for new and more accurate benchmark datasets for statistical microarray analysis. We present here a fresh method using biologically-relevant data to evaluate the performance of statistical methods.

**Results:**

Our novel method ranks the probesets from a dataset composed of publicly-available biological microarray data and extracts subset matrices with precise information/noise ratios. Our method can be used to determine the capability of different methods to better estimate variance for a given number of replicates. The mean-variance and mean-fold change relationships of the matrices revealed a closer approximation of biological reality.

**Conclusions:**

Performance analysis refined the results from benchmarks published previously.

We show that the Shrinkage *t *test (close to Limma) was the best of the methods tested, except when two replicates were examined, where the Regularized *t *test and the Window *t *test performed slightly better.

**Availability:**

The R scripts used for the analysis are available at http://urbm-cluster.urbm.fundp.ac.be/~bdemeulder/.

## Background

### Objectives

The sensitivity of microchip data analysis tools is strongly limited by the weakness of the estimation of variance because the number of replicates is generally low and variance heterogeneity is high. Several methods, variants of the classical *t *test [1], have been developed in recent years to increase this sensitivity by improving the estimation of variance. These methods are generally benchmarked on artificial ("spike-in") or simulated data. Consequently, the ability of the methods to better estimate variance is tested only on technical or modelled variances, and not on biological variance. We propose to evaluate these statistical strategies on actual biological data in order to avoid this bias. As the use of actual data does not allow for definition of the unambiguous "truth" to identify true and false positives, we propose a novel approach to circumvent this limitation.

### State of the art

Microchip data analyses are confronted with the double-edged problem of multiple testing and weak variance estimation due to the often limited number of replicates. Furthermore, departure from normality and variance heterogeneity between genes and between experimental conditions for a given gene can decrease the confidence of statistical tests. Moreover, data has shown that a non-trivial mean-variance relationship benefits to methods analyzing groups of genes [2,3] instead of analyzing genes separately. This relies on the fact that n genes sharing similar expression levels also share more similar variances than n genes sampled randomly.

Aside from the classical Welch correction for variance heterogeneity [4], numerous heuristics have been developed over recent years to improve the estimation of variance and consequently the statistical power of the tests. The Window *t *test, Regularized *t *test and LPE test [2,3,5] assume an empirical relationship with the average expression level. SAM, the Regularized *t *test and Moderate *t *test (Limma) use an empirical Bayes model to estimate the variance [2,6,7]. The Moderate *t *test and two versions of a shrinkage approach base the estimation of variance on distributional assumptions [7-9].

Table 1 shows the variance shrinkage used in different heuristics (Regularized *t *test, SAM, Moderate *t *and Shrinkage *t*). The general formulation of the variance estimator can be written: V = a V_0 _+ b V_*g*_. Thus, the variability estimator is estimated from two terms, respectively background variability V_0 _and individual variability V_*g*_. Those terms are first used either to compute a sum (SAM) or a weighted average value (Regularized *t *test, Moderated *t*, Shrinkage *t*). This is operated at the variance level, except in the SAM procedure where the offset term is added at the level of the standard deviation.

**Table 1:** Summary of variance shrinkage used by several procedures

Method	Equation	Shrinkage level	Offset Estimation
Regularized t-test		*V *= *S*^2^	Window Estimator

SAM d-statistic (Tusher)	*a *= 1 *b *= 1	*V *= *S*	*S*_0 _= arg min(*CV*(*MAD(d*_*a*_))) Fudge factor minimizing CV

Moderated-t (Limma)		*V *= *S*^2^	*ψ*, *ψ' *= *digamma, trigamma functions*

Shrinkage-t	*b *= 1 - *a*	*V *= *S*^2^	

The general formulation V = a V_0 _+ b V_g _illustrates that the variability estimator is estimated from two terms, respectively the background variability V_0 _and individual variability V_g_.

In the Regularized *t *test procedure, an arbitrary parameter is used to weight this mean value, based on the number of replicates (n + n_0 _= k = arbitrary value = 10 in the original procedure, where n is the number of replicates and n_0 _is the number of "virtual replicates" used to compute the background variance). Limma also uses the degrees of freedom associated with each variability term as weighting factors. The degrees of freedom associated with background variance are computed from the expression data matrix, considering a mixture of genes with and without differential expression.

To compute the Shrinkage *t *statistic, those terms are weighted according to the minimum value between one and an intermediate statistic reflecting the dispersion of individual estimates compared with their deviation from the median value.

Another diverging aspect of the procedures is estimation of the correction term used to shrink variance towards an optimal value. The background term is computed using different procedures: (i) from a relationship between expression level and variability (Regularized *t *test), (ii) from the value minimizing the dispersion of the Student *t *derivate statistic (SAM), (iii) from a mathematical model describing the mixture of two sets of genes (Moderated *t*), and from the median value of the individual variance distribution (Shrinkage *t*).

Using their own variance estimates, each method computes a *t *statistic, either based on equal variances (SAM, Moderated *t*, Shrinkage *t*) or unequal variances (Regularized *t *test, Shrinkage *t*). The significance of each statistic is then assessed by comparison with a null distribution, in accordance with the model: (i) the *t *distribution with degrees of freedom computed following Welch's correction (Regularized *t *test), (ii) from the cumulative distribution associated with the 2 sets of genes, with corrected degrees of freedom (Moderated *t*), or (iii) empirically from permutations of sample labels (SAM). The Shrinkage *t *procedure does not include a null distribution and only uses the *t*-like statistic to rank the results.

The differences between the statistics thus lie in the way in which the variances and/or their degrees of freedom are computed. Paradoxically, the datasets available to compute the rates of false positives and negatives and thus evaluate the sensitivity and specificity of each approach are based on simulated or spike-in data. Such data is characterized by variances, variance heterogeneities and mean-variance relationships which differ from those actually observed with biological data. The problem when benchmarking these methods is precisely the discrepancies between the data used and the performances allegedly tested.

### Existing benchmark datasets

Over recent years, we have witnessed the emergence of a huge number of pre-processing and processing methods for microarray analysis. To validate these approaches and compare their performances, we need datasets for which both non differentially-expressed genes and truly differentially-expressed genes (DEG) are known. Up to now, this question has been addressed by spike-in experiments or in silico simulations.

#### a - Spike-in datasets

A spike-in experiment is a microarray experiment where RNA is added in known quantities. A few datasets of this type are available, namely the two Latin Square datasets from Affymetrix [10] and the "golden spike" experiment [11]. These datasets were used in several papers to compare methods that analyse differential expression in microarray experiments [12-15]. However, the results appear to be highly dependent on the dataset chosen to test the methods, which can be explained by the extremely divergent characteristics of these datasets.

The Affymetrix Latin Square datasets are characterized by a very low number of differentially-expressed genes (42/2230 genes, about 0.2% of all genes, in the HG-U133 Latin Square), an extreme fold-change range (from 2 to 512) and a large concentration range (from 0.125 pM to 512 pM in the HG-U133 Latin Square). In these datasets, a complex human RNA mixture (human cRNA from the pancreas in HG-U95) was added under all experiment conditions to mimic the bulk of non differentially-expressed genes.

Choe's spike-in dataset [11], made with a Drosophila chip, was designed to compensate for the failings of existing datasets, and differs considerably from the Latin Square datasets on a number of points: (i) the proportion of spiked DEGs is high, about 10% of all genes; (ii) RNA was spiked in high quantities (iii) only up-regulated genes were included in the dataset, which is not expected in real experiments; (iv) no unrelated background RNA was used, but an important number of genes were spiked in equal quantities on all arrays. This made it possible to distinguish between empty genes, and genes expressed with no differential expression. In Affymetrix's Latin Squares, the complex and undefined background RNA eliminated the possibility to distinguish between unexpressed and expressed genes.

The aim of spike-in datasets is to mimic a typical microarray experiment, and their main problem is determination of parameters such as the proportion of DEGs and their concentration, the up- or down-regulation of genes, the amount of the mixture that is added to mimic the bulk of equally-expressed genes. However, these parameters influence the results as well. For example, the proportion of DEGs influences the normalization procedure, which assumes that the majority of genes are not differentially expressed, but it cannot be defined from actual experiments where this proportion remains unknown. Each one of the two available types of spike-in datasets has dramatic biases, and re-analyses have been performed on Choe's dataset to take them into account [12,13,15].

Performances can be compared together on both datasets considered as two extreme but imperfect conditions. Then the "best" combination of pre-processing and processing would be that which provides the best performance in both tests. However, this pragmatic approach does not lead to an improved understanding of the underlying mechanisms and parameters which make a method perform better than another under given conditions. Moreover, biological variance is not taken into account, as both datasets contain only technical replicates.

#### b - Simulation datasets

Some authors have tried to model in silico microarrays. Among others, Parrish *et al *[16] and Singhal *et al *[17] attempted to model the complex reality of a microarray on the basis of observation or real datasets.

The first study was based on a multivariate normal distribution (by selecting mathematical transformations of the underlying expression measures such that the transformed variables approximately follow a Gaussian distribution, and then estimating the associated parameters) in order to model transformed gene expression values within a subject population, while accounting for co-variances among genes and/or probes. This model was then used to simulate probe intensity data by a modified Cholesky matrix factorization [16].

Though Singhal's general approach might appear to be similar to ours as it is also based on real datasets, his method differs in the fact that he extracted parameters (biological and technical variance) from these datasets to simulate datasets based on the parameters [17], while we use the data itself. Like all simulated datasets, numerous simplifications are made and skew reality. So, Parrish *et al *approximated Gaussian distributions and Singhal *et al *approximated biological and technical variance using mathematical equations, which inevitably skews or impoverishes reality.

In conclusion, in a traditional spike-in dataset (Affymetrix Latin Squares and Golden Spike experiments), over-expression is simulated by the addition of RNA fragments at known concentrations, with great reproducibility between the replicates [13]. Important biological variability observed in real datasets is completely eliminated. When the truth is simulated in silico [16,17], classical biases generated by the simplification of modeling are expected.

The classification of statistical methods thus reflects their ability to detect true positives and avoid false negatives in an artificial context, which is in obvious contradiction with the fact that methods differ primarily in their approach to the estimation of variance. The reliability of these benchmarks is thus open to discussion at the very least. A biological microarray dataset for which the truth is known simply does not exist.

## Methods

### Strategy proposed

The goal of our approach was to benchmark different statistical methods on authentic biological data in order to preserve the actual mean-variance relationship. The "truth" is not inferred by simulation or induced by spike-in of a known concentration of genetic material. Different sets of genes are defined as the truth, designed to be more or less difficult to isolate from the background.

We selected the genes from archived experiments on at least 15 replicates under two experimental conditions on one same platform (Affymetrix's HG-U133a). This number of replicates represented a good compromise between dataset availability and variance estimation quality. Indeed, when n = 15, the difference between the Z and *t *distributions is very slight.

The "truth" is defined as a set of genes characterized by a predetermined ratio between differential expression and variability between replicates. This ratio is computed such that under optimal conditions (normality, homoscedasticity and known variance) the classical *t *test would be characterized by a given sensitivity and a given positive predictive power (see below: theoretical background). The sensitivity and positive predictive power are then fine-tuned to render genes increasingly difficult to distinguish from the background. The capability of the various statistical methods to detect these sets of genes is then tested on a limited subset of replicates selected at random from among those used to define "the truth".

Thus, the benchmark does not compute false positive and negative rates in comparison with an experimentally-validated "truth" - which is unrealistic - but tells that, if the "truth" were to be this set of genes, the performances of the methods would be those evaluated.

Several problems are circumvented using this approach such as: (i) the fundamental problems of respect of actual biological variance, the respect of the dependence of this variance on the level of gene expression, the difference in variance between genes as well as between control and test replicates are addressed by collecting actual experimental data, (ii) the prevalence of differentially-expressed genes, often limited in spike-in data (0.2% DEGs in the Latin Square datasets), is controlled and kept constant by re-sampling in over 1,000,000 DB-probesets (we call one row of the DB matrix a "DB-probeset" to avoid confusing with an original probeset (in any classic expression set)) obtained through the combination a large number of datasets, (iii) uneven detection efficiency due to a mix of extreme fold change in a same benchmark (from 2 to 512 in the Latin Square datasets) is avoided by defining more homogeneous differentially-expressed sets of genes. This means that methods are evaluated for a given detection limit and not for a mix of genes in which some are trivial and some are too difficult to detect.

Finally, a non-trivial problem addressed here is that the variation of the number of replicates influences the statistical power both through variance estimation quality and the magnitude of the standard error. We fine-tuned the ratio between differential expression and variability according to the number of replicates (n) (the higher the n, the lower the ratio) so that the difficulty to find a set of genes considered as the truth would remain constant if the variance were known. The effect of n on variance estimation quality can thus be strictly isolated and improvements in the estimation of variance can be evaluated in detail.

### Theoretical background

The positive predictive power (PPP) of a test is defined as a function of the numbers of true positives (TP) and false positives (FP) according to Equation 1.(1)

Let P be the prevalence of over- or under-expressed genes, α the probability of type I error and β the probability of type II error. Equation 1 can be transformed to express PPP as a function of (1-β), α and P (Equation 2) and, from there, α as a function of (1-β), P and PPP (Equation 3) [18].(2)

Let n be the number of replicates (considered constant along a procedure), σ^2 ^the variance considered as homogeneous and μ_0 _- μ_1 _the difference between gene expression under conditions 0 and 1. n can then be expressed as a function of power (1-β) and confidence (1-α) according to Equation 4 [18].(4)

Let D = M_0_-M_1_, the estimation of μ_ο_-μ_1_of variance 2 σ^2 ^and S the estimation of σ. The ratio D/S_threshold _expressed in Equation 5 is directly related to the ability of the Student *t *test to detect a given differential expression on n replicates with power (1-β) and confidence (1-α).(5)

As D/S_threshold _is computed from 15 replicates, it provides a rather good estimate of μ_0 _- μ_1 _and σ. It approximates the limit for rejecting Ho under ideal conditions (normal distribution and homoscedasticity) with the Student *t *test at a given n, power and confidence.

Two main qualities of a test may be considered to be its sensitivity (1-β) and positive predictive power (PPP) (Equation 1 and 2). When high, these probabilities ensure that the user will find an important part of the truth, with low random noise, respectively.

In our benchmark, we fixed the number of replicates for subsets of replicates (n), prevalence (P), sensitivity (1-β) and positive predictive power (PPP). The value of α is deduced from Equation 3 and the corresponding D/S_threshold _computed from Equation 5. This allows us to define a subset of genes which is more or less easy to detect from the background, and to keep this difficulty constant when increasing n to improve the quality of the variance estimate.

### Implementation

Data collection (*cel files*) was performed using Gene Expression Omnibus [19] on the Affymetrix platform HG-U133a (Human Genome model U133a). This collection consists of 34 datasets (table 2) for which there are at least 15 replicates for each of 2 different experimental conditions. With all the *cel files *from one experiment, we built an Affybatch object, which is simply a structured concatenation of the files. These 34 Affybatch objects were pre-treated using the R package GCRMA [20]. As the benchmark is tested gene by gene, a pre-treatment including all Affybatch objects globally was not needed.

**Table 2:** Datasets list

Dataset	Number of replicates
GSE10072	**107**

GSE10760	**98**

GSE1561	**49**

GSE1922	**49**

GSE3790FC	**65**

GSE3790CN	**70**

GSE3790CB	**54**

GSE3846	**108**

GSE3910	**70**

GSE3912	**113**

GSE5388	**61**

GSE5392	**82**

GSE5462	**116**

GSE5580	**42**

GSE5847	**95**

GSE646-7	**93**

GSE643-5	**126**

GSE648-9	**125**

GSE650-1	**122**

GSE6613	**105**

GSE7670	**53**

GSE7895	**104**

GSE8401	**83**

GSE8835	**65**

GSE8397	**47**

GSE9676av-ap	**60**

GSE9676m-f	**60**

GSE9676m	**30**

GSE9676f	**30**

GSE9716	**38**

GSE9874b-f	**60**

GSE9874	**60**

GSE9877	**47**

GSE994	**75**

Datasets used for construction of the initial matrix. The number of replicates is the number of microarrays in the experiment.

Giant datasets (e.g., GSE3790 with 202 replicates in three different brain regions) were first split into subsets according to their biological content. The datasets were then sampled as follows: when the number of replicates was ≤ 29, 15 replicates were selected randomly. When the number of replicates was ≥ 30, 15 replicates were selected randomly a first time, and a second time in the remaining replicates, and so on for 45, 60 replicates or more.

**Figure 1:**
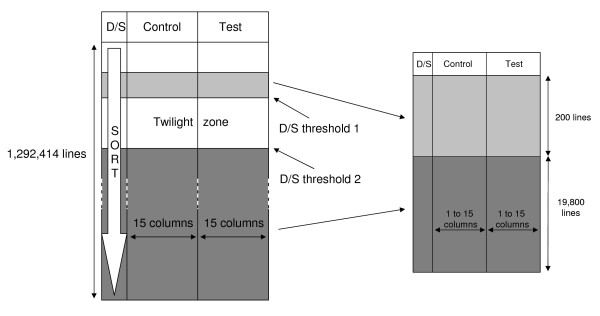
**General sample design**. Left panel: matrix DB, appending 34 datasets. Computation of two D/S_threshold _ratios (see text) determines different zones in the matrix. The light grey zone contains DEGs and is integrated to the subset matrix (right panel) as such. The dark grey zone contains the non DEGs. 19,800 rows are selected randomly within this zone to form the background of the subset matrix. A twilight zone segregates the DEGs from the non DEGs.

The resulting "Expression sets" were appended in a single matrix (named DB below) of 2 × 15 columns (replicates) collecting 1,292,414 lines (DB-probesets). The D/S ratio was computed for each DB-probeset, where D is the difference between the means and S is the square root of the mean variances under the two experimental conditions. The matrix DB was sorted according to the |D/S| value, from the top corresponding to the most over- or under-expressed genes (relative to their standard error) to the bottom corresponding to non differentially-expressed genes (figure 1).Subset matrices were sampled randomly 5 times from DB as follows: the dimensions were set at 20,000 DB-probesets and 2 × n replicates to correspond roughly to an actual expression set (figure 1). The prevalence which was defined at 1% (200 DB-probesets) is a compromise between enough genes to accurately compute frequencies of true and false positives and not too much to get a relative homogeneity of D/S in the set. Incidentally, this prevalence is in the order of magnitude of current lists of expected genes of interest in many biological contexts.

For a relative evaluation of the statistical methods, the D/S_threshold _was moved according to the increase of n, such that the difficulty to find a set of genes considered as the truth would remain constant if the variance were known. For a given set of parameters n, PPP and (1-β), the D/S_threshold _was computed and the 200 genes above the limit were selected in DB and considered as the target genes (true positive). A second D/S_threshold _was computed to correspond to 0.9 × (1-β) and 19,800 genes considered as the background (the true negative) were selected randomly in DB, under this limit. The genes in the "twilight zone" between the two limits were not considered to avoid an abrupt transition between both gene statuses.

Finally, to evaluate the absolute performance of the best statistical methods, the D/S threshold was computed for n = 2 and for given combinations of PPP and (1-β), thus defining the "truth" constant for every set of subset matrices for the run considered; five subset matrices of 15 replicates - instead of two - were generated as describe above and resampled for n = 2, 3, up to 10 such that the difficulty to find a set of genes considered as the truth increased according to n, due to the combined effect of improved variance estimation *and *reduced standard error.

Each subset matrix was treated using the PEGASE software developed in our laboratory (Berger *et al*., CEJB, under revision). Briefly, several differential expression analysis methods were implemented from scratch and gathered in the R package called PEGASE. Among the methods currently implemented for differential expression analysis are the classic Student *t *test [1] and Welch correction for heteroscedasticity [4], SAM [6], Regularized *t *test [2], Window *t *test [3]. The package includes a performance evaluation of the methods implemented when a list of truly differentially-expressed genes is provided. Limma [7] and Shrinkage *t *[9] are not yet implemented in PEGASE and were downloaded and run stand-alone.

For each combination of parameters and statistical analysis, we computed the observed power, or sensitivity (Equation 6) and the false positive rate (Equation 7) for five samples for increasing values of α, step by step.(6)

An ANOVA with 3 fixed criteria (statistical methods, n and runs) was run over the five random samplings of replicates in DB, on the sensitivity computed for 1% FDR. This ANOVA produced a residual mean square (RMS) value corresponding to the error term of each fixed effect. This RMS was used in post-hoc comparisons performed for pairwise comparisons of the methods [21] and for comparisons of each method with the reference method [22].

### Algorithm

The input used in the algorithm which computed the performance curve coordinates (FDR, Sensitivity, and Specificity) was the full list of p values for each method. As each list of p values does not cover the same range of values, we needed to use the minimum significance value to define the starting point of the procedure. Moreover, as the beginning of the curves is the most informative, corresponding to small p values, we decided to define each step from regular progression at the logarithm of p values. The pseudo-code of the algorithm used is described below:

1) Retrieve the minimum p value (min.pval);

2) Compute the logarithm of this minimum value (log.min.pval), with base = 10;

3) Compute log.int = vector with 1000 values defining regular intervals between log.min.pval and 0 (corresponding to the maximal p value = 1);

4) Compute the final list of values defining the intervals (int), using int = 10^log.int;

5) For each value in int, compute FDR, sensitivity, specificity from each list of method-specific p values.

## Results

### Mean - Standard deviation relationship

**Figure 2:**
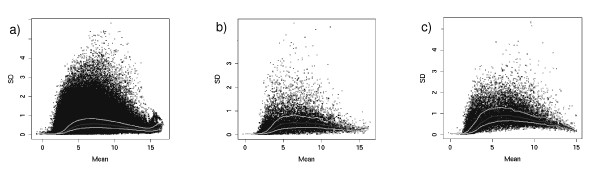
**Mean versus sd plots**. Standard deviation versus mean expression level. a) total benchmark dataset; b) subset of the total biological benchmark dataset; c) biological dataset (EMEXP-231). The dark grey curves represent the medians of the values and the light grey curves represent quartiles (median ± 25%).

Figure 2 illustrates the empirical relationship between the average expression level and standard deviation. Figure 2A represents the relationship observed for the total benchmark dataset. Figure 2B shows the corresponding plot obtained from a subset of the total biological benchmark dataset. Finally, figure 2C presents an example of the same graph generated on a biological dataset (E-MEXP-231 from Array Express) [23,24] which was not included in the creation of the benchmark dataset.

Interpreted together, the plots shown in figure 2 reveal that the design of the Biological Benchmark from real datasets (2 A) leads to a similar expression level/variability dependence compared with real datasets (2 C). The definition of subsets based on the D/S statistic combined with the positive predictive value and power parameter generates datasets with properties which are similar to real datasets (2 B).

### MAplot

**Figure 3:**
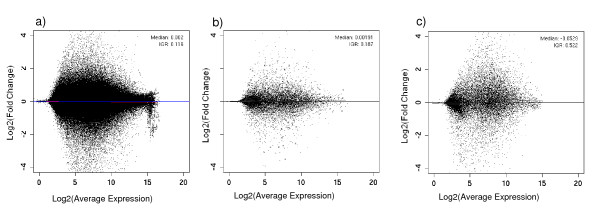
**MAplots**. A MAplot represents the average log expression versus the average ratio (or fold change) between two conditions. a) total benchmark dataset; b) subset of the total biological benchmark dataset; c) biological dataset (EMEXP231).

A MAplot represents the average log expression versus the average ratio between conditions. Figure 3C shows the MAplot obtained from the same dataset used for figure 2[23], after pre-processing (GCRMA). As can be seen in the figure, points are typically widely distributed along the X-axis while being centered around M = 0 on the Y-axis. Figures 3A and 3B show the MAplots from our total benchmark dataset and a subset thereof. The similarity between these two figures and figure 3C highlights that our dataset distributions are close to a real dataset distribution. In comparison, MAplots obtained from spike-in datasets clearly show their biases, especially the extreme fold changes of the Latin Square LS95, and the absence of down-regulated DEGs in the Golden Spike Experiment dataset (LS95, LS133: [10,11]).

### Volcano plots

Different sets of parameters were tested and those retained here are the most typical, intermediate values which provide intermediate results. Four runs were performed for increasing difficulties to find the target DB-probesets. For run 1 (PPP = 0.99 and sensitivity = 0.99), the true positives were easy to find and there was little noise. For run 2 (PPP = 0.5 and sensitivity = 0.99), the true positives were easy to find and there was more noise. For run 3 (PPP = 0.99 and sensitivity = 0.5), the true positives were harder to find but there was less noise. And for run 4 (PPP = 0.5 and sensitivity = 0.5), the true positives were difficult to find and there was more noise.

**Figure 4:**
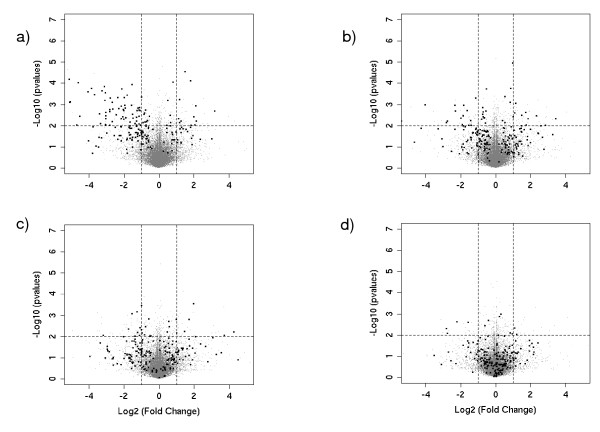
**Volcano plots**. Volcano plots show the -log_10 _(p-values) versus the log2(fold change). In black, the DEGs, in grey, the non DEGs. The horizontal line represents the 10^-2 ^threshold on the p values, while the vertical lines show thresholds of ± 2 fold changes. From a) to d) (runs 1 to 4), the difficulty of finding DEGs increases.

Volcano plots present the DB-probesets in a graph of p values according to a given statistical test versus fold change. In the present context, they represent the increasing difficulty to find true positives through runs (figure 4). Typically, interesting features are located in the upper left and right corners of the graphs, as the fold change values (X axis) and p values (Y axis) exceed the usual thresholds used for analysis.

Volcano plots were drawn for the four runs with the Student *t *test with three replicates. On 200 DEGs, in run 1, nearly one half (92) of the target DB-probesets (black points) had a p value lower than 10^-2 ^and the average fold change was -1.19 ± 4.1 (M ± 2 s.d.). As expected, in run 2, fewer target DB-probesets were found to be significant (44) and the average fold change was -0.29 ± 3.82. In run 3, most of the target DB-probesets did not exceed the significance threshold of 10^-2 ^(29) and the average fold change was -0.147 ± 3.44. Finally, under the most difficult conditions (run 4), only few target DB-probesets still exceeded the statistical thresholds (12) and the average fold change was -0.006 ± 1.96. Surprisingly, as revealed by the negative mean value, most of DEGs were down-regulated, but this fact will not have an impact on our results.

### Relative performances of the statistical methods

**Figure 5:**
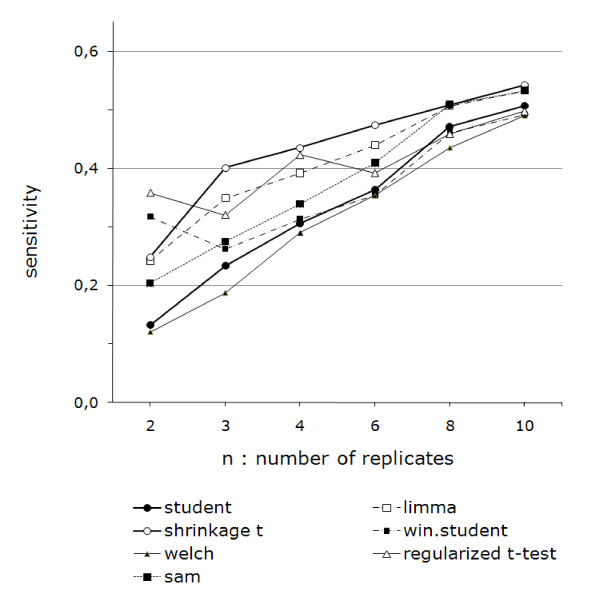
**Relative performances of the methods**. Relative performances of the methods. Sensitivity versus number of replicates for statistical methods (GCRMA as pre-treatment, run 2, FPR and prevalence = 1%). Except for two replicates, the Shrinkage t performs the best. For 10 or more replicates, SAM and the Regularized t test are as efficient.

**Figure 6:**
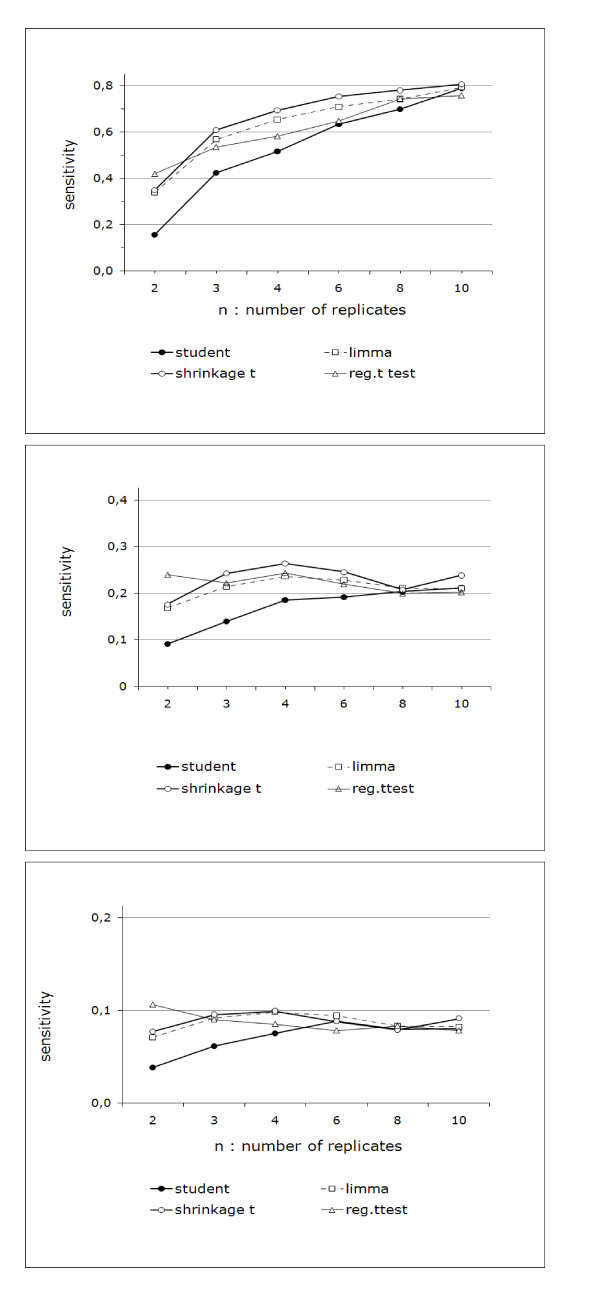
**Sensitivity versus number of replicates**. Sensitivity versus number of replicates for classic Student t test, Shrinkage t, Limma and Regularized t test. From top to bottom: run 1, run 3 and run 4 (DEGs increasingly difficult to find). Except for two replicates, Shrinkage t performs the best. For two replicates, Regularized t test performs best.

Figures 5 and 6 show the change in sensitivity for all (figure 5) or a selection (figure 6) of the methods studied. They present a summary of the information from all of the ROC curves for all n, taken at a FPR equal to prevalence (1%). This value was chosen because ROC curves become non-informative when the FDR exceeds the prevalence [25]. For more details, see additional file 1.

Run 2 (figure 5) is illustrated for all of the methods tested. As the Shrinkage *t *and Limma on one hand and the Regularized *t *test and Window *t *test on the other showed only slight differences between them, only a selection of methods is displayed for purposes of clarity in figure 6 for Runs 1, 3, and 4, with the Student *t *test as the reference.

Though it has become obvious that the Shrinkage *t *is most often the best method, we show that, when there are only two replicates, the Regularized *t *test and Window *t *test are better. We also observed that when they are 10 or more replicates, the choice of the method becomes less important, as all of the methods perform quite equally.

A ranking of the methods is presented in table 3 for all runs and number of replicates (rank 1 being the best). We only show statistical differences found by Dunnet's post hoc comparison with p ≥ 0.05. We could not highlight any significant difference for run 4. Across all of the statistically relevant data, the Shrinkage *t *appears to have the lowest mean ranking. It shall therefore be considered as the reference method from now on. However, we noted a striking change between 2 and 3 or more replicates: the Regularized *t *test and Window *t *test appear to have the best performances only for n = 2.

**Table 3:** Ranking of the performances

RUN 1							
*n*	*2*	*3*	*4*	*6*	*8*	*10*	*Average*
Student	7	7	5	5	5		5.8
Window	**2**	6	7	7	7		5.8
Welch	8	8	6	6	6		6.8
Win Welch	5	5	8	8	8		6.8
Reg t test	**1**	3	4	4	3		3.0
SAM	6	4	3	3	**2**		3.6
Limma	4	**2**	**2**	**2**	4		2.8
Shrinkage t	3	**1**	**1**	**1**	**1**		1.4
RUN 2							
*N*	*2*	*3*	*4*	*6*	*8*	*10*	*Average*
Student	7	7	6	5	4		5.8
Window	**2**	6	5	6	5		4.8
Welch	8	8	8	7	7		7.6
Win Welch	3	5	7	8	8		6.2
Reg t test	**1**	3	**2**	4	6		3.2
SAM	6	4	4	3	**1**		3.6
Limma	5	**2**	3	**2**	3		3.0
Shrinkage t	4	**1**	**1**	**1**	**2**		1.8
RUN 3							
*n*	*2*	*3*	*4*	*6*	*8*	*10*	*Average*
Student	8	8	5				7.0
Window	**2**	5	6				4.3
Welch	7	7	8				7.3
Win Welch	3	4	7				4.7
Reg t test	**1**	**2**	**2**				1.7
SAM	6	6	4				5.3
Limma	5	3	3				3.7
Shrinkage t	4	**1**	**1**				2.0

Ranking of the performances of all the methods tested. Only significant differences (DUNNET'S post hoc comparison p ≥ 0.05) for each method with respect to Shrinkage t are displayed (no significant difference in Run 4). 1^st ^and 2^nd ^ranks are shown in bold.

### Absolute performance

To assess the absolute performances of the methods tested, we performed a new test such that the difficulty to find a set of genes considered as the truth increases according to n, due to the combined effect of improved variance estimation *and *reduced standard error (see Implementation).

The ranking of the three methods presented is the same as before: the Shrinkage *t *is better overall, except for n = 2, where the Regularized *t *test (superimposed to Window *t *test, data not shown) is slightly better. Moreover, this figure presents the maximal performances of the methods with respect to the run considered, as the truth defined for two replicates is the easiest to recover.

For Shrinkage *t*, in a gene list where 1 false positive is expected for 1 true positive, 80% of sensibility is expected for the run 1 with n = 3 when the fold change is -1.234 ± 4.42, for the run 2 with n = 4 when the fold change is -0.812 ± 4.5, for the run 3, with n = 7 when the fold change is -0.46 ± 3.86 and for run 4 with n > 10 when the fold change is -0.013 ± 2.3.

## Discussion

Our benchmark dataset is difficult to objectively compare with previously published benchmarks because we used a different approach, where the definition of the truth was not so straightforward and irrefutable but actual variance was conserved.

It is probable that use of the D/S ratio to infer the truth introduces a bias towards *t*-like statistics. This is why we only measure performances for such methods. As for methods in specific, we think that this bias does not change their ranking.

For example, the Limma method may be favored by this bias as it relies on the existence of two different distributions (DEG and non-DEG) and the benchmark creates those two distributions using a twilight zone. Thus, the Limma method's performances should be better than those of Shrinkage *t *test, globally based on the same principles but not using a pre-defined distribution. However, we show that the Shrinkage *t *performs slightly but significantly better than Limma.

In the Golden Spike Experiment [11], the authors compared the Regularized *t *test with the Student *t *test and SAM. The relative ranking of the methods was comparable with our results (several methods tested here were not published then). In the original Limma paper [7], among other results, the authors showed that Limma performs better than the Student *t *test. This conclusion was in keeping with our findings. In the original Shrinkage *t *paper [9] the authors ranked the methods as follows: Shrinkage *t *similar but in some cases better than Limma better than Student better than SAM. For us, SAM performs better than Student, but not as well as the Shrinkage *t *and Limma.

Finally, in Berger's paper [3], we showed an advantage for methods adapted to better estimate variance, and in particular for the methods using a window to define the target genes. Here, we show that the Window *t *test and Regularized *t *test perform equivalently, however not as well as the Shrinkage *t *and better than Limma, except notably when the number of replicates is two.

All of the previously published results are in accordance with the results presented here, as we show that the methods based on either shrinkage of the window variance estimator (Shrinkage *t *test, Regularized *t *test and Window *t *test) provide the best performance. However, we can affirm that our results are more representative since they were obtained from analysis of actual biological data.

As pointed out in a recent reanalysis of the Golden Spike experiment [12], spike-in datasets available to date, while valuable, either contain too few DEGs, or are flawed by several artifacts, such as unrealistically high concentrations of DEGs. In conclusion, the authors encouraged the creation of new spike-in datasets in order to complete and improve the method for benchmarking of DEG analysis of Affymetrix assays. Such datasets should have the following characteristics: (1) a realistic spike-in concentration, (2) a mixture of up- and down-regulated genes, (3), unrelated fold change and intensity, and (4) a large number of arrays.

Here, we propose a dataset that is not a spike-in dataset, though we believe that it meets the conditions stipulated in the article by Pearson.

Several studies (e.g. [2,3]) on differential expression analysis have postulated a complex relationship between variability and expression level. In some methodologies [2,3,5], this empirical relationship was used to improve the assessment of variance in a statistical framework. Spike-in and simulated datasets do not take this empirical relationship into account, compared with the biological benchmark described in this paper. The relationship found in our data (figure 2) reveals that the design of our biological benchmark from real datasets leads to a similar expression level/variability dependence compared with real datasets.

Many factors can influence the variability of expression of probesets, from technical sources to biological properties, and simulation of realistic variance components is not completely straightforward. Genes present both shared and diverging properties. In this context, creation of a benchmark dataset from a repository of biological datasets preserves individual variability properties, as no assumptions on individual variance are needed during the creation of the benchmark dataset. Each potential source of variation is retrieved from real data, thus retaining the contributions from sources of variability, without the need to quantify or list them.

The MAplots of our datasets show that the genes which we defined as DEGs are present at all concentrations, with variable fold change (1) and meeting point (3). Selected genes were shown to be a mixture of up- and down-regulated genes, meeting point (2). Finally, we performed analyses using a number of replicates going from ten to two by condition, meeting point (4).

We have shown with the mean versus standard deviation relationship, MAplots and volcano plots that the datasets we built are closer to real datasets in terms of expression and fold-change distribution, than those of spike-in datasets such as the Latin Square HGU95 and HGU133a from Affymetrix or Choe's Golden Spike Experiment. Moreover, the resulting dataset contains biological as well as technical variability and we have shown that it is representative of the mean-variance relationship of real datasets.

ROC curves were only used in this work to generate the data used to construct figures 5 and 6. We used the values for a FPR equal to the prevalence, as, above this limit, the number of false negative exceeds the number of positives (see additional file 1 for details).

These figures present the core benchmarking results. They reveal that, among the methods tested, the Shrinkage *t *test performs best under all conditions (number of replicates and difficulty to find the truth), although when the number of replicates is very low (< 3), the Regularized *t *test and Window *t *test show slightly better performances and when the number of replicates is high (≤ 10), the choice of the method has a lower impact on performance. The reason why the Shrinkage *t *does not perform well for two replicates is that it does not rely on a pre-defined distributional model. This implies that it needs several replicates to assess this distribution. The fact that the Window *t *test and Regularized *t *test take the number of replicates into account in the statistic calculation is the reason why they perform better when the number of replicates is low.

**Figure 7:**
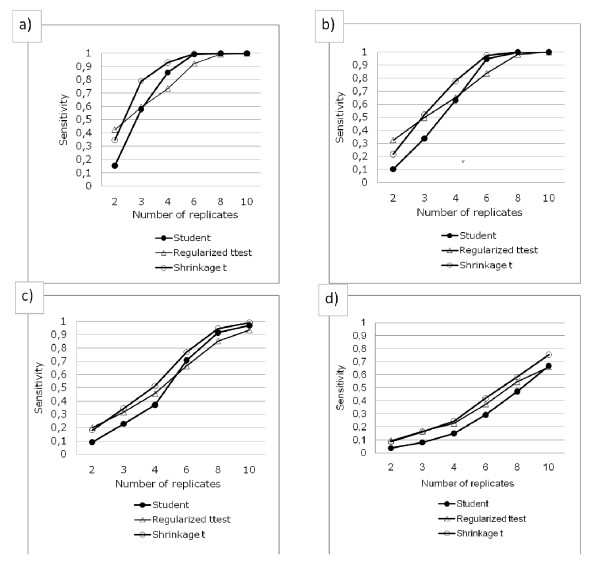
**Absolute performances of the methods**. Absolute sensitivity versus number of replicates for classic Student's t test, Shrinkage t and Regularized t test. From a to d: Runs 1 to 4 (DEGs increasingly difficult to find). As previously commented, the Shrinkage t performs best overall, except for 2 replicates.

We then computed the absolute performances of three methods. The results presented in figure 7, although limited to one-color arrays under GCRMA as pre-treatment, confirm the trends which we suggested with relative results (figure 5 and 6). Keeping in mind that, in some pathways, even slight differences in gene expression can lead to dramatic changes in terms of metabolic effects, one should be aware that the methods tested here, although among the best available today, could still be greatly improved.

One could thus raise the question as to the reliability of the results when the number of replicates is low. One way to address this issue would be to adapt the methods to better estimate variance when the number of replicates is low. Another way would be to perform statistical analysis on relevant groups of genes rather than on isolated genes. The design of relevant groups of genes still remains a challenge.

## Conclusions

The benchmark method proposed here differs from other approaches published, as actual biological and experimental variability is preserved. The obtained Mean - Standard deviation relationships and MAplots confirm that the variance structure of the data we studied is closer to biological data than that of spike-in or simulation studies. One other advantage of the method lies in the fact that virtually all parameters can be fine-tuned, allowing researchers to assess those methods which are truly suited for their particular approaches.

We applied the benchmark to a set of published methods. The results show better performances for the Shrinkage *t *test, except when there are only two replicates, where the Regularized *t *test and Window *t *test perform better.

## Perspectives

In order to compare all the analytical methods, including pretreatments, we also plan to modify the way in which the truth is defined in our DB matrix, for example using an *in silico *spike-in procedure and finding a way to preserve the biological variances associated with the DB-probesets. However this constraint is not trivial to circumvent.

In this study, we only work with GCRMA as the pretreatment, with a prevalence of 1%. Some authors [26] show that correlations between probesets can also influence performances of the statistical methods, namely favoring the Shrinkage *t *and Limma. In the future, our work will concentrate on an exhaustive study of the nested effects of those three parameters (pretreatment, prevalence and correlation), but is outside the scope of this paper due to its complexity. In the same way, we could improve the way we present the results by using a classification based on the level of expression for example.

## Authors' contributions

BDH. took part in designing the method we present here, as well as in interpreting results. BDM. scripted the whole methodology, apart from the PEGASE package. He also ran the analysis and took part in designing and interpreting the results. FB scripted the PEGASE package and took part in graphical representation of the results. MP analyzed and interpreted the volcano plots and related data. EB took part in scripting and in collection of the data. AG analyzed and interpreted the MA plots and related data. ED coordinated the whole work and gave final approval for submission. Each author read and approved this manuscript.

## Supplementary Material

Additional file 1 **Supplementary data**. Parameterization. ROC curve analysis details. Figures 5, 6 and 7 with error bars. Calculation of the number of rows used throughout all the analysis. Click here for file
