# Supplementary material for: A benchmark for statistical microarray data analysis that preserves actual biological and technical variance
Source: BMC Bioinformatics. 2010 Jan 11;11:17. doi: 10.1186/1471-2105-11-17 (PMC2831002; doi:10.1186/1471-2105-11-17)
Supplement: Additional file 1 — Supplementary data. Parameterization. ROC curve analysis details. Figures 5, 6 and 7 with error bars. Calculation of the number of rows used throughout all the analysis. [file 1471-2105-11-17-S1.DOC]

###### Supplementary data

##### Parameterization

Figure A is a visual representation of the different runs (1 to 4) explained in the main text.

Figure A.

**ROC curve analysis**

ROC (Receiver Operating Curve) curves are widely used in the field of microarrays. When working on datasets where the truth known, the ROC curve is defined as a plot of the proportion of modified genes truly found (sensitivity) as the y coordinate versus the proportion of genes falsely found as the x coordinate (1-specificity or false positive rate (FPR)).

The use of ROC curves originates from radar detection assays during the Second World War. They were further adopted in the medical field to highlight the relationship between sensitivity (proportion of persons having a disease that are detected positive) and specificity (proportion of healthy persons that are detected negative). However, in the case of rare pathologies, a diagnostic test characterized by both good (>95%) sensitivity and specificity can exhibit very low positive predictive values (PPV), meaning that a person being positive to the test is more likely to be healthy.

This last consideration could be expressed in terms of the number of true positives (TP) and false positive (FP):

- If we accept that 1% prevalence (about 200 genes out of 20,000) is a plausible gene list in a biological context, and if we imagine 100% sensitivity (TPR) (200 TP found), 1% FPR (200 FP found) means a ratio of 1 TP for 1 FP in the gene list.

- If we consider that 10% prevalence (about 2000 genes out of 20,000) is a more reasonable gene list, and if we imagine 100% sensitivity (TPR) (2000 TP found), 10% FPR (2000 FP found) means the same ratio of 1 TP for 1 FP in the gene list.

- If sensitivity falls to 50% (100 TP found) - a value currently observed in benchmarks - a 1% FPR means a ratio of 1 TP for 2 FP in the gene list, and so on.

Figure B.

For us, this means that over a FPR above prevalence, the ROC curves are non-informative (the very beginning of the ROC x-axis) and biological interpretation of the gene list is nonsensical (figure B).

Basically, study of figure C shows that if the truth is more difficult to find (from runs 1 to 4), the performances of the methods decrease. Likewise, if the number of replicates falls (from 10 replicates to 3), the performances will be worse. But we can also say that when we look at a ROC curve over the whole range of FPRs, it is impossible to discriminate between the performances of the methods for every set of parameters used in the analysis. Correspondingly, a zoom in on the meaningful part of the curve (see below) shows significant differences between the performances of the methods.

Figure C.

Moreover, we have shown that the interesting part of the curve lies below an FPR equal to the prevalence of the analysis. It is thus mandatory to zoom in on this interesting part. The zoomed figures (figure D) show indeed that when using the part of the curve below the prevalence, one can more easily discriminate between the performances of the methods.

Figure D.

A look at figure D (showing the ROC curves for an FPR between 0 and 10%) confirms the comments made for figure 2 about the general performances of the methods. From runs 1 to 4 and from 10 to 2 replicates, performance decreases.

Study of the left-end part of the curve (where FPR <= prevalence) highlights the best methods for the particular set of parameters used. We can see the best methods are the Shrinkage t, Regularized t test or Window t test, depending on the parameters used.

Finally, we present the mean fold change values +- 2 SEM, for all of the runs with 3 replicates in table I. This shows that there were no significant differences between the different values; however, we show a decrease in the mean fold change as the difficulty increases (Runs 1 -> 4), which is consistent with the methodology used to define the truth.

**Figures with error bars**

Figure E, F and G.

As proposed by the reviewers, we present here the figures 5, 6 and 7 of the main article with error bars. We think the adding of error bars complicates the reading without adding much information. Nevertheless, we present them here for completeness’ sake.

**Calculation of the number of row used throughout all the analysis**

DEG part: we sample 200 rows, for 24 different conditions (6 number of replicates and 4 runs): 200 * 24 = 4,800 rows

Background part: we sample 19,800 rows for 24 conditions 5 times :

19,800 * 24 * 5 = 2,376,000 rows.

Thus, in total we analyze 2,380,800 rows.

We can thus say that, although only a part of the results obtained are extensively discussed for clarity, we actually used a lot more data than what appears.


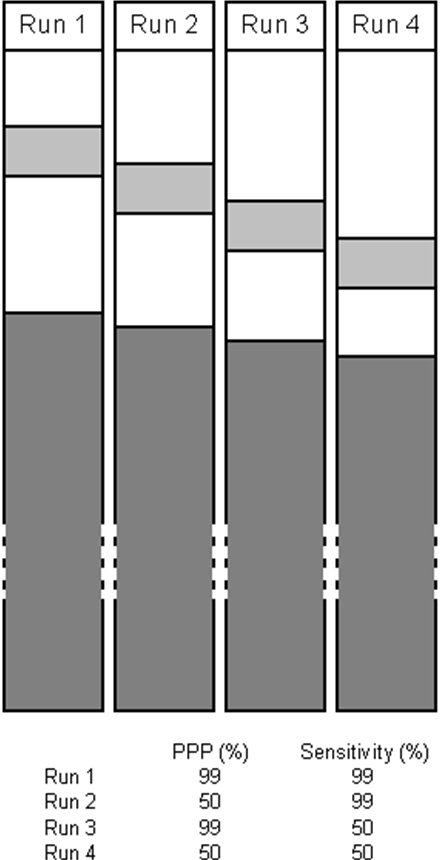


*Figure A. The increasing difficulty from run 1 to run 4 derives from two sources: a) decrease of the D/S_threshold_ ratio, which makes the DEG “window” (shown in light grey) drop and b) the decrease of the width of the twilight zone (shown in white). The dark grey zone contains the non DEGs.*

*Figure B. Artificial curves that show a sensitivity (TPR) of 100% (a) and 50% (b). The dashed black lines correspond to a FPR of 1% and the grey lines to a FPR of 10%. Transforming the percentages into the number of probesets (see the text) clearly shows that over a FPR higher that the prevalence, ROC curves are non-informative.*

*
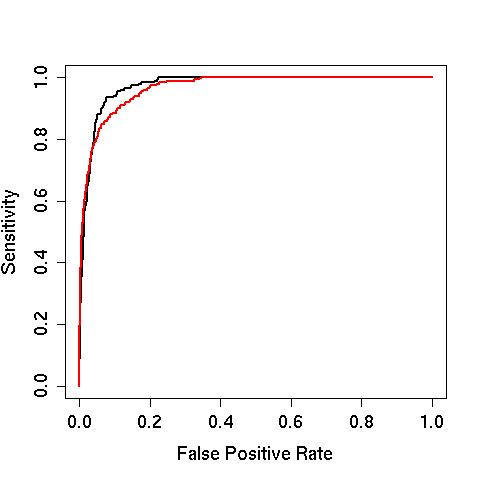

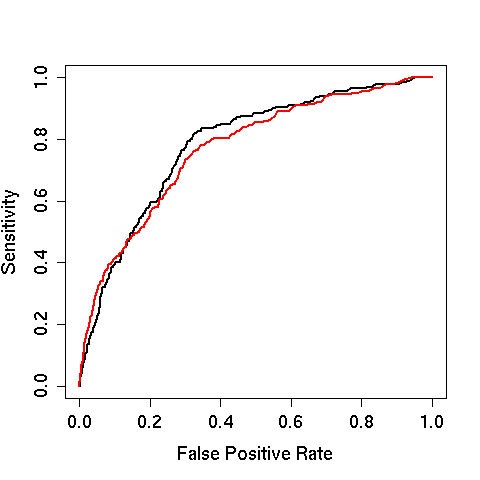

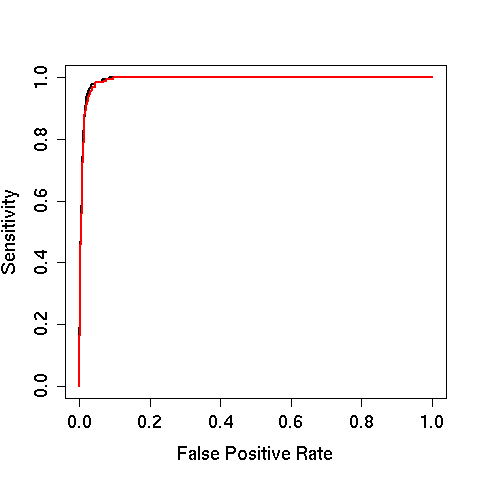

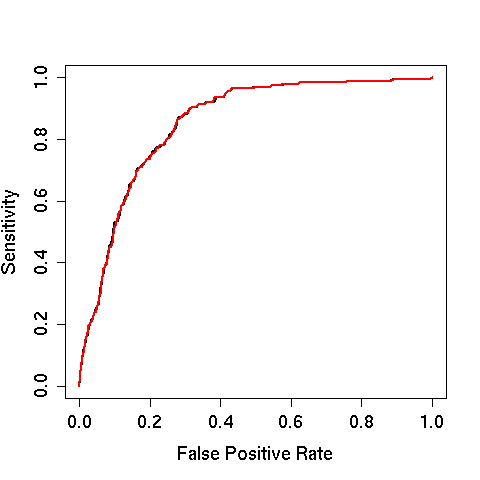
*

a)

b)

c)

d)

Figure C. ROC Curves generated from our benchmark for the Student t test and Shrinkage t. The four combinations of parameters used were: a) run 1 and 10 replicates; b) run 1 and 3 replicates; c) run 4 and 10 replicates; d) run 4 and 3 replicates. Black curves: Student t test; grey curves: Shrinkage t.


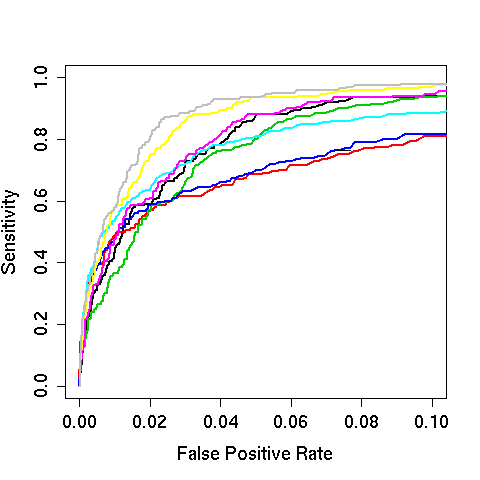

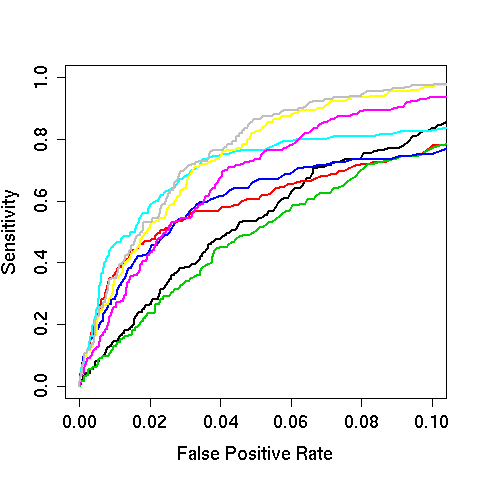

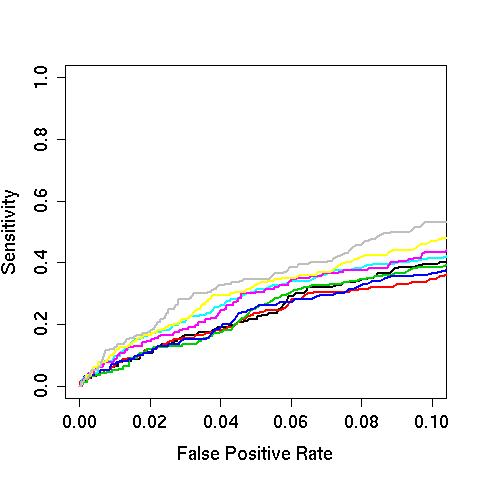

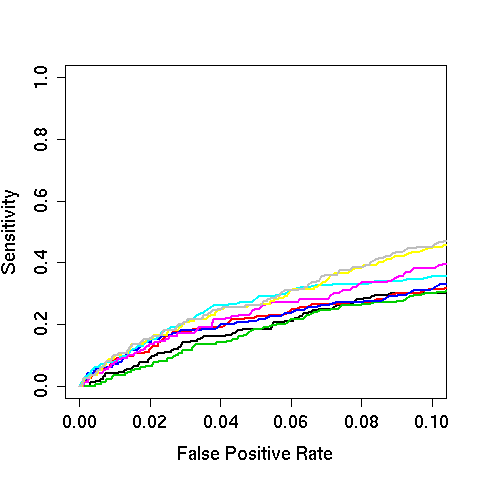


a)

b)

c)

d)


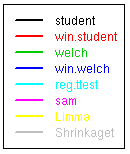

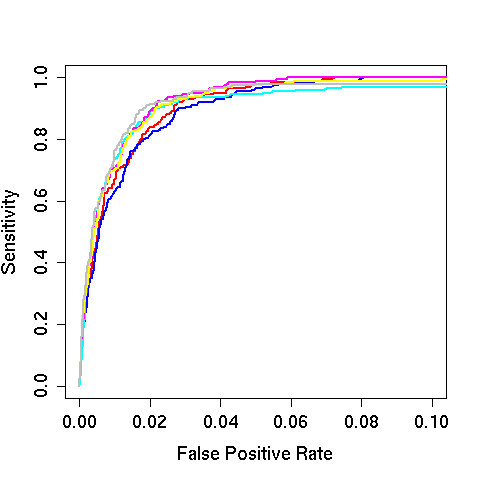

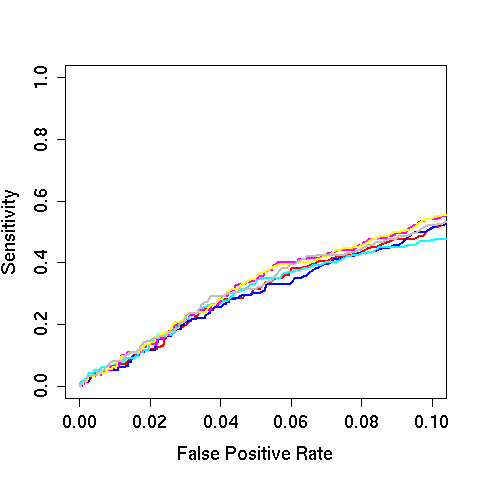


e)

f)

Figure D. Roc Curves generated from our benchmark for eight “Student-like” methods. As we are working on a prevalence of 1%, only the left sides of the curves (FPR <0.01) are really informative, the number of false positives at this percentage being already higher than the number of positives. Parameters used: a) run 1, 2 replicates; b) run 4, 2 replicates; c) run 1, 3 replicates; d) run 4, 3 replicates; e) run 1, 10 replicates; f) run 4, 10 replicates.

Table I: Fold change values +- 2 SEM for all runs with 3 replicates.

|  | RUNS | | | |
| --- | --- | --- | --- | --- |
|  | 1 | 2 | 3 | 4 |
| Mean | -1.190 | -0.290 | -0.147 | -0.006 |
| Mean + 2SEM | 2.910 | 3.534 | 3.293 | 1.954 |
| Mean - 2 SEM | -5.290 | -4.114 | -3.587 | -1.966 |


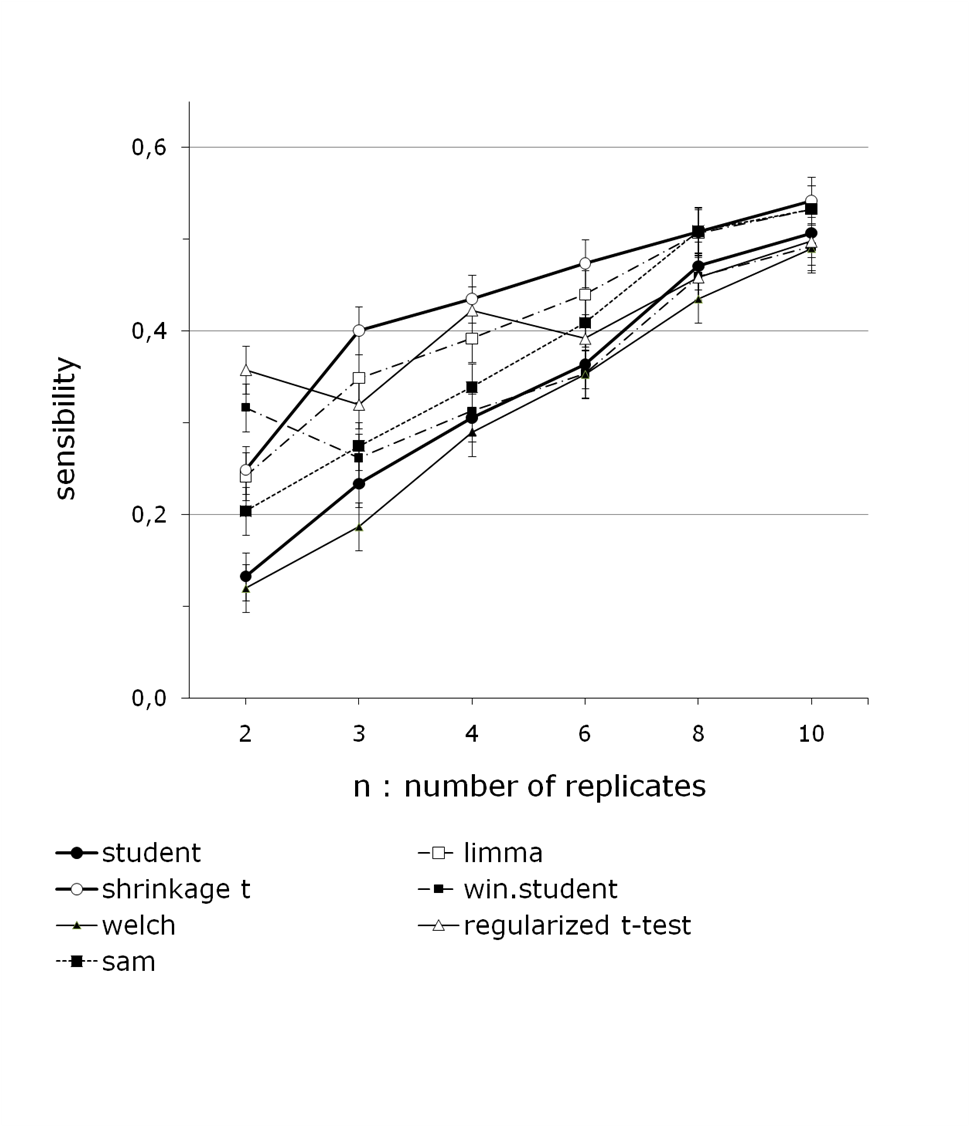
 Figure E. This figure is the same as the figure 5 in the main text, except this one has error bars (+ - 2 SE). One standard error = 0,013.


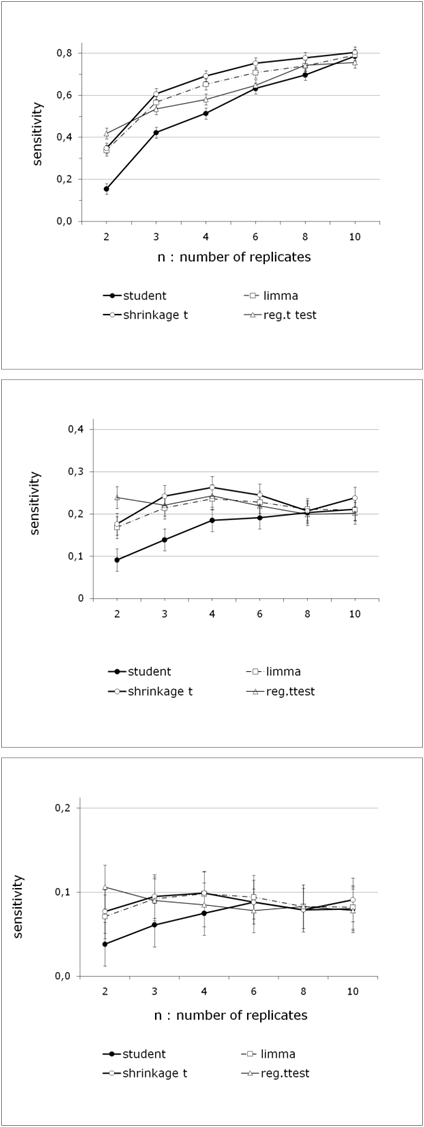
 Figure F: This figure is the same as figure 6 in the main text, except this one has error bars (+ - 2 SE). One standard error = 0,013.


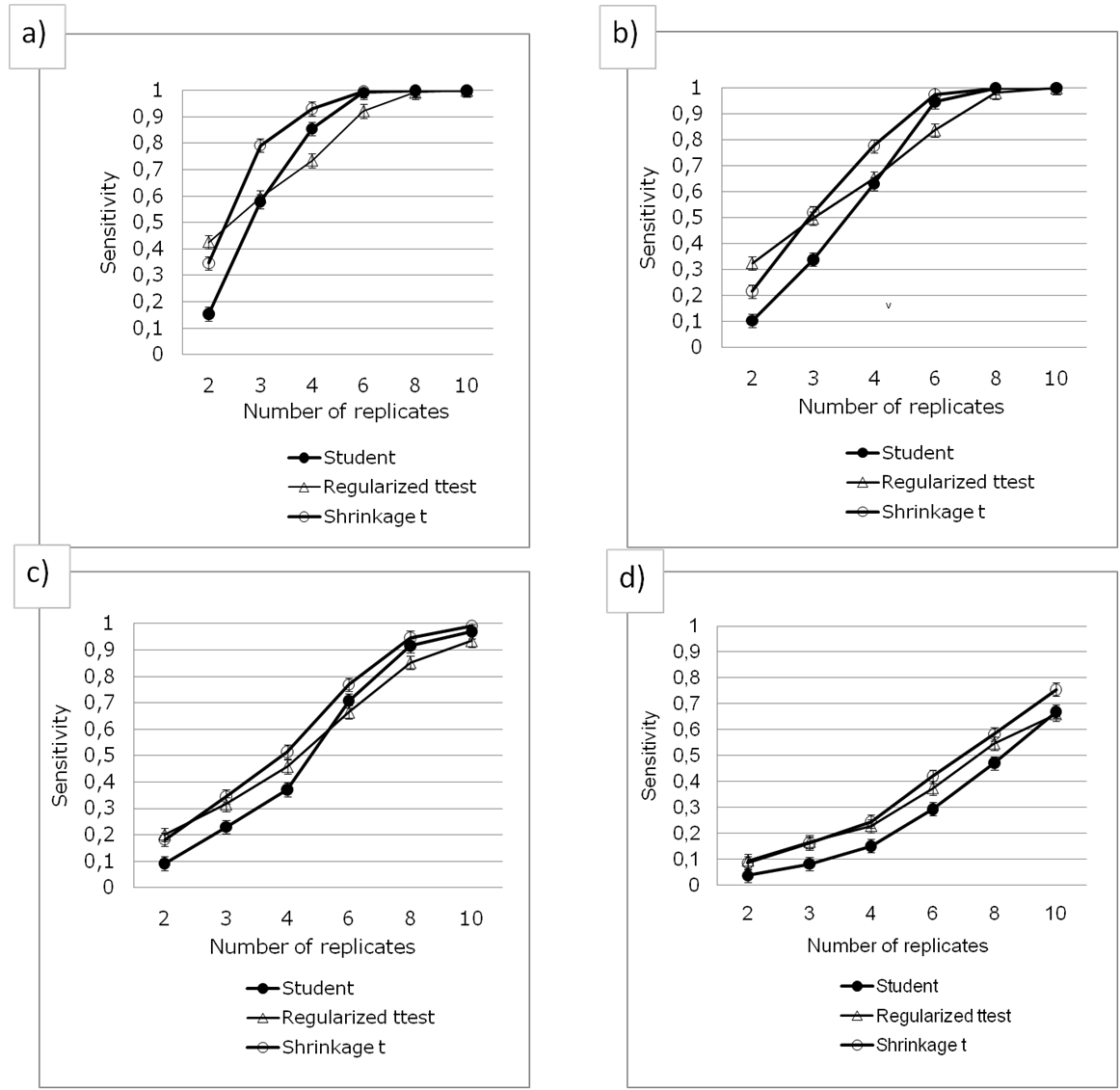


Figure G: This figure is the same as figure 7 in the main text, except this one has error bars (+ - 2 SE). One standard error = 0,013.
